# Macrophages in microgravity: the impact of space on immune cells

**DOI:** 10.1038/s41526-021-00141-z

**Published:** 2021-03-31

**Authors:** Christopher Ludtka, Justin Silberman, Erika Moore, Josephine B. Allen

**Affiliations:** 1 J. Crayton Pruitt Family Department of Biomedical Engineering, University of Florida, Gainesville, FL USA; 2 Materials Science and Engineering, University of Florida, Gainesville, FL USA

**Keywords:** Acute inflammation, Chronic inflammation

## Abstract

The effects of a microgravity environment on the myriad types of immune cells present within the human body have been assessed both by bench-scale simulation and suborbital methods, as well as in true spaceflight. Macrophages have garnered increased research interest in this context in recent years. Their functionality in both immune response and tissue remodeling makes them a unique cell to investigate in regards to gravisensitive effects as well as parameters of interest that could impact astronaut health. Here, we review and summarize the literature investigating the effects of microgravity on macrophages and monocytes regarding the microgravity environment simulation/generation methods, cell sources, experiment durations, and parameters of interest utilized within the field. We discuss reported findings on the impacts of microgravity on macrophage/monocyte structure, adhesion and migration, proliferation, genetic expression, cytokine secretion, and reactive oxygen species production, as well as polarization. Based on this body of data, we make recommendations to the field for careful consideration of experimental design to complement existing reports, as the multitude of disparate study methods previously published can make drawing direct comparisons difficult. However, the breadth of different testing methodologies can also lend itself to attempting to identify the most robust and consistent responses to microgravity across various testing conditions.

## Introduction

During extended time during spaceflight, astronauts experience significant changes to their health and immune system^1^. When immune system functionality is altered, the ability to recognize antigens, defend against foreign invaders, and orchestrate repair are significantly hindered. This alteration in immune functionality leads to an increased risk of infection, however, how spaceflight impacts immune and health functionality is still unknown^2,3^. To expand the field of human spaceflight, scientists seek to understand the specific mechanisms through which immune functionality is changed and develop countermeasures for these effects. Many of the functions of the immune system are largely monitored and regulated by immune cells known as monocytes/macrophages. Monocytes and macrophages are present in all tissues and are critical for initial host immune responses, tissue development, homeostasis, and tissue repair^4^. To learn more about how the immune system is affected in spaceflight, monocytes/macrophages are key targets of microgravity experiments^4^.

In this review, we evaluate the study of monocytes and macrophages in microgravity over the past 30 years. Due to the recent emergence of models in this area, we believe now is a valuable time to review the state of the field. Through this review, we aim to provide a central hub for current methods and findings on monocytes/macrophages in microgravity. To start, we discuss conventional methods and devices used to study microgravity. We then analyze the current research efforts towards elucidating the macrophages changes that occur in microgravity. We conclude with closing remarks highlighting commonalities across the breadth of research and provide some perspective on where the field is headed.

## Microgravity Models

Cellular research under simulated and true microgravity has revealed a variety of biological processes that are significantly impacted by changes in gravity. While a great deal has been learned, much remains unknown, in part due to the scarcity of prolonged spaceflight opportunities caused by their associated costs and logistics. As such, several ground-based or alternative strategies have been developed to simulate or achieve microgravity. Ground-based simulators allow researchers to gather preliminary data before space experimentation. Several comparative studies and reviews have been written to validate, compare, contrast, and define many different microgravity experimental methods^5–7^.

**Fig. 1:**
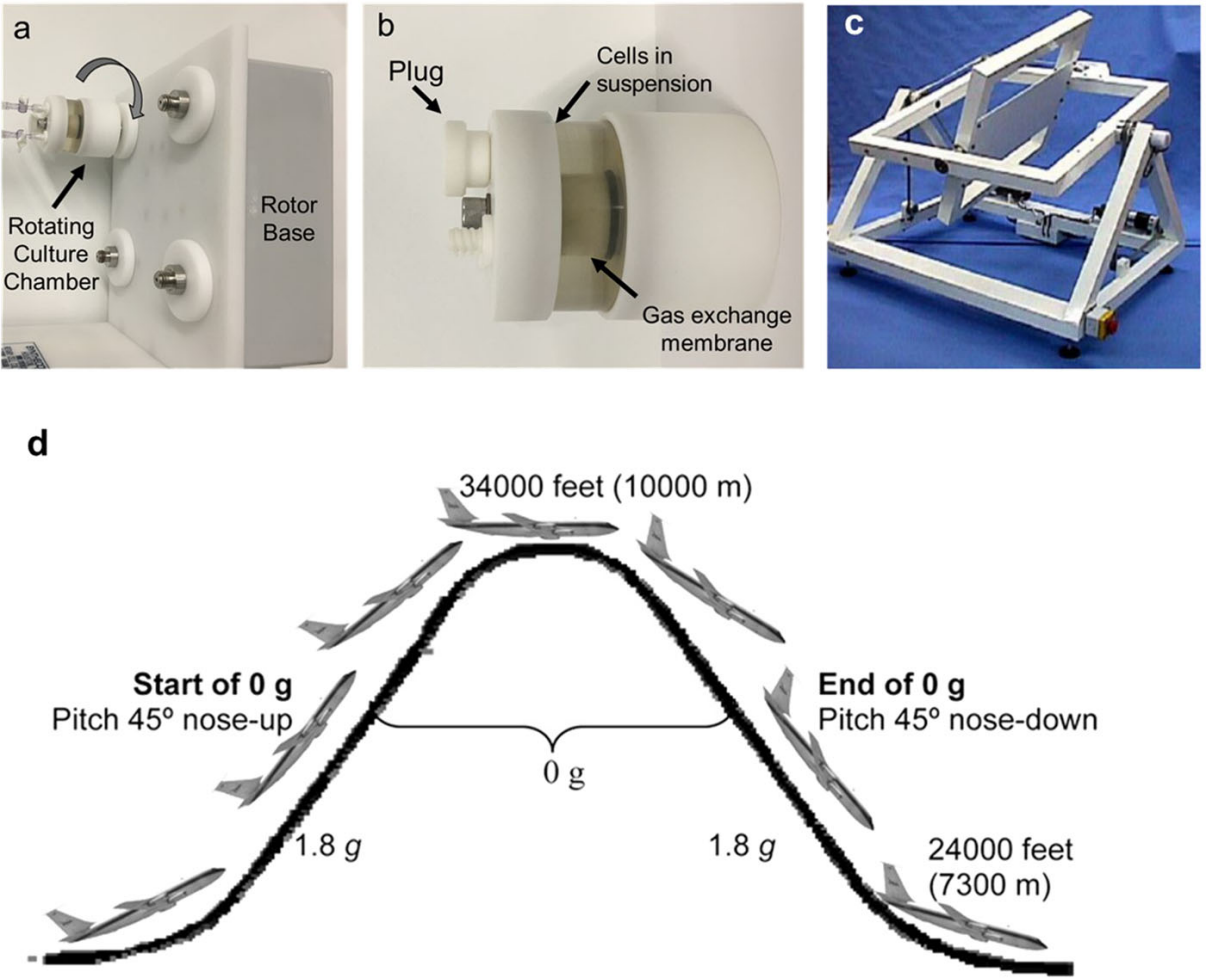
Representative examples of experimental methods used to simulate microgravity. Panels **a**, **b** show digital images of a rotating wall vessel (RWV) bioreactor. Here vascular cells are grown in culture chambers which are then rotated concentrically around a single axis. Panel **c** shows a RPM with two independently driven perpendicular frames^83^. Panel **d** shows the parabolic flight trajectory^84^ used to create short-duration microgravity exposure.

The primary methods of simulating microgravity in cell culture and methods used in many of the manuscripts included in this review are two-dimensional or three-dimensional (2D or 3D) cell culture through 2D or 3D clinorotation. Fundamentally, clinorotation changes cells’ perception of Earth’s gravitational pull, from constant 1-g towards Earth to a consistently changing direction during rotation of cells in culture^7^. 2D clinorotation rotates cells at a particular speed that matches the sedimentation rate of the cells in culture. At the correct speed, cells are in continuous “free-fall” through the media, thus simulating weightlessness. An example of this system is NASA’s rotating wall vessel (RWV) (Fig. 1a, b), in which cells are in 3D culture while aggregated on microcarrier beads^8^. 3D clinorotation is achieved using random positioning machines (RPM) (Fig. 1c), which rotate samples on two separate frames such that the average direction of the gravity vector is near zero. For the methods mentioned, there are variations in the number of rotation axes, speed, and direction of rotation depending on the sample.

Parabolic flight and sounding rockets are also used as a method for microgravity studies with periods of 20–30 s and 4–5 min of weightlessness, respectively. These methods utilize a parabolic flight pattern which induces periods of microgravity (Fig. 1d). Each device, strategy, or method used to simulate or achieve microgravity has some limitations—including exposure to hypergravity, microgravity duration, and altered culture conditions—that must be considered when interpreting simulated microgravity studies. While these methods are common and have advanced the field of microgravity studies, it should also be noted that studies in simulated microgravity are strengthened by correlative studies in space that validate these methods and the corresponding results^9^.

## Monocytes and Macrophages

Monocytes, commonly known as precursors to macrophages, originate from the bone marrow and circulate through the blood. As a part of the inflammatory immune response, monocytes circulating in the blood adhere to the endothelium and extravasate towards tissues in need of immune intervention. During and after monocyte migration towards their destination, both macrophages and monocytes produce many pro-inflammatory cytokines such as tumor necrosis factor alpha (TNF-alpha), interleukin 1 (IL-1), IL-6, IL-8, and IL-12 to further promote the immune response and recruit more inflammatory cells^10^. Alongside their adhesion functionality and other responsibilities in the immune response, monocytes most notably differentiate into macrophages^11^. Macrophages are significant players in the initiation, propagation, and resolution of inflammation. Macrophages undergo phenotypic differentiation into “classically activated” M1 macrophages, which are pro-inflammatory and “alternatively activated” M2 macrophages, which are pro-healing. The M1 phenotype is induced in vitro with common factors associated with pathogen recognition such as lipopolysaccharide (LPS), zymosan, or IFN-γ, which increase phagocytosis, production of ROS, and other inflammatory behaviors^4^. M2 is induced by stimulation with IL-4, IL-10, and IL-13, which causes a decrease in antigen presentation and an increase in receptors associated with debris clearance^12^. For simplicity, macrophage phenotype is kept binary (M1, M2) in many in vitro experiments reviewed here, however, important work has shown that there is a spectrum of activation levels in macrophages with less distinct properties in vivo^13^. M1 and M2 macrophages vary in their gene expression, surface marker expression, and cytokine secretion profiles, which results in wide-ranging cellular functions and behaviors. In this review, we focus on aspects of macrophage biology under investigation in these microgravity studies. Excellent, comprehensive reviews of normal macrophage biology and function in health and disease are presented elsewhere, particularly in the context of the vasculature^14–16^. As spaceflight has a notable effect on astronaut vasculature^17,18^ and vascular endothelial cells are also affected by exposure to microgravity^19–21^, these evaluations are particularly relevant.

## Microgravity Effects on Macrophages

Not surprisingly, cells of the immune system are particularly susceptible to microgravity conditions^22–26^. This may be due to their unique functionality regarding inflammation and stresses within the body or their reliance on blood circulation, which can be altered in the body by microgravity conditions^18^. Based on numerous studies evaluating aspects of macrophage biology under the effect of real and simulated microgravity, macrophages demonstrate many sensitivities to changes in gravity, both in their short and long term responses^22^. However, given the differences between the cell sources used in studies (described in Table 1), as well as the microgravity platforms, and experimental variables such as microgravity duration, it is difficult to directly compare studies and challenging to interpret results^27^. Table 2 outlines the various experimental platforms, cell types, durations, and results in the literature. Collectively, however, microgravity (real and/or simulated) is shown to induce changes in macrophage metabolism, signal transduction, proliferation, cytokine secretion, differentiation, cytoskeletal structure, gross morphology, locomotion, gene expression, and inflammatory response^28–30^. Any number of these changes can contribute to an altered immune response while in space.

**Table 1:** Summary of major macrophage cell sources used in microgravity experiments.

Cell type	Description
Primary macrophages/monocytes	Cells harvested and isolated from living tissue samples, such as blood or immune cell-rich organs (e.g., bone marrow, spleen)
RAW 264.7 cells	Murine macrophage-like cell line from an Abelson murine leukemia in BALB/c mice
U937 cells	Human leukemic myelomonocytic cell line that differentiates into a macrophage-like phenotype
NR8383 cells	Rat alveolar macrophage cell line isolated from lung tissue
B6MP102 cells	Murine bone marrow-derived macrophage factor-dependent cell line
J-111 cells	Human acute monocytic leukemia cell line, but contaminated with HeLa cells (HPV-related endocervical adenocarcinoma)

**Table 2:** Outline of experimental platforms, cell types, durations, and results in the literature of macrophage microgravity research.

Experiment platform	Paper	Cell type	Time in µg	Results
Clinorotation (simulated µg)	Shi 2020	Primary mouse	12 d	Macrophage differentiation from hematopoietic progenitor cells and functional polarization impaired in µg; RAS/ERK/NFκB pathway gravisensitive
	Wang 2015	Primary mouse	24 h	Simulated µg activates p38 MAPK-C/EBPβ pathway; increased arginase expression; upregulated IL-6, downregulated IL-12B
	Brungs 2015	NR8383	4 h	Reduced ROS production in µg; diminished Syk phosphorylation; NFκB nuclear translocation unaltered
	Paulsen 2015	BV-2 microglial cells; U937; Primary human	1 d; 3 d; 5 d	Reduced ICAM-1 expression in BV-2 cells; increased ICAM-1 expression in U937 and primary human macrophages
	Wang 2014	RAW 264.7; Primary mouse	24 h	LPS-induced TNF-α expression in µg reduced, but not IL-1β; increased HSF-1 in µg; nuclear translocation of NFκB unchanged
	Adrian 2013	NR8383	30–180 m	ROS release is reduced in µg, responding rapidly and reversibly
	Paulsen 2010	U937	5 m	Non-stimulated macrophages had enhanced tyrosine phosphorylation and c-jun activation; PMA-stimulated macrophages had reduced tyrosine phosphorylation and c-jun activation
	Maier 2006	U937	24 h; 72 h	Upregulation of hsp70; inhibited proliferation; altered cytokine secretion in µg
	Hsieh 2005	RAW 264.7	2 d	Reduction in nitric oxide and cytokine production; flavonoids compensated for loss of macrophage function in 3D culture
	Maccarrone 2003	U937	2–72 h	No expression of active 5-LOX or apoptosis, in contrast to lymphocytes
	Hashemi 1999	Primary human	24 h	Normal IL-1 synthesis
Random position machine (simulated µg)	Meloni 2006	J-111	1 h; 24 h	Cell motility severely reduced in µg; actin, tubulin, and vinculin structures affected
Limb suspension (simulated µg)	Liu 2015	Primary rat	28 d	µg increased monocyte recruitment, E-selectin and MCP-1 expression, and NFκB activation in abdominal aorta
Suborbital/sounding rocket (real µg)	Thiel 2019	Primary Human	353 s	µg induces cell geometry changes, rapid cytoskeleton reorganization, and rapid adaptation of the cytoskeleton
	Vogel 2019	U937	300 s	HIF-1 differently regulated in altered gravity; some genes have rapid response and adaptation, others altered only after 5 min. in µg
	Thiel 2018	U937	300 s	Transcriptome greatly altered in µg; 99.43% of all initially altered transcripts adapted after 5 min.
	Paulsen 2015	U937	378 s	No detectable effect on ICAM-1 mRNA expression
Parabolic flight (real µg)	Vogel 2019	U937	20 s	HIF-1 differently regulated in altered gravity; some genes have rapid response and adaptation, others altered only after 5 min. in µg
	Thiel 2018	U937	20 s	Transcriptome greatly altered in µg; 99.43% of all initially altered transcripts adapted after 5 min.
	Paulsen 2015	BV-2 microglial cells; U937; Primary human	20 s	Rapid, reversible downregulation of ICAM-1 in BV-2 cells; upregulation of ICAM-1 in differentiated U937 cells; Primary human inconclusive
	Adrian 2013	NR8383	22 s	ROS release is reduced in µg, responding rapidly and reversibly
	Paulsen 2010	U937	20 s	1.3-fold increased MEK phosphorylation; rapid p53 phosphorylation in non-stimulated U937 cells
	Armstrong 1995	B6MP102	20 s	Macrophages respond to µg within 8 s, with increased cell spreading
Spaceflight (real µg)	Shi 2020	Primary mouse	12 d	Macrophage differentiation from hematopoietic progenitor cells and functional polarization impaired in µg; RAS/ERK/NFκB pathway gravisensitive
	Tauber 2017	Primary human	11 d; 30 d	Decreased ICAM-1 and surface-bound fucose; CD18 and CD14 surface expression unaltered
	Thiel 2017	NR8383	15 m	Immediate oxidative burst inhibition followed by extremely rapid adaptation to µg
	Paulsen 2015	U937	5 d	Upregulation of ICAM-1 in differentiated U937 cells
	Paulsen 2014	U937	5 d	Disturbed actin, disorganized tubulin, and reduced CD18, CD36, MHC-II expression in µg
	Crucian 2011	Primary human (blood sample)	13–16 d	Inflammatory phenotype and cytokine production impacted by spaceflight; LPS-stimulated cells showed reduced IL-6, TNF-α, IL-10, and increased IL-1b in µg
	Meloni 2011	J-111	24 h	Severe reduction in motility attributed to disruption of cytoskeleton; actin, tubulin, vinculin distribution affected
	Baqai 2009	Primary mouse (spleen sample)	13 d	Spaceflight can increase anti-inflammatory mechanisms; altered response to LPS stimulation; reduced monocyte/macrophage count; upregulated ROS genes post-flight
	Kaur 2005	Primary human (blood sample)	5–11 d	Monocytes had reduced phagocytic ability, oxidative burst, and degranulation after spaceflight
	Hatton 1999	U937	9 d	Kinetics of protein kinase C are modified during spaceflight.
	Chapes 1999	Primary rat (peritoneal sample)	10 d	Enhanced secretion of TNF, IL-6, and nitric oxide
	Hashemi 1999	Primary human	22 h; 25 h	Normal IL-1 synthesis
	Armstrong 1995	B6MP102	6–8 d	Increased macrophage proliferation; reduced IL-6 secretion; reduced differentiation
	Chapes 1992	B6MP102	6 d; 9 d	Greater secretion of IL-1 and TNF of LPS-stimulated macrophages in µg

*µg* microgravity, *s* second, *m* minute, *h* hour, *d* day.

### Structural effects

Macrophages are large cells that have well-characterized structural features, which correspond with many of their functional roles. For example, resting macrophages have irregular cell borders and pseudopodia pushed out in all directions^31^. Activated M1 macrophages have their actin cytoskeleton arranged throughout the cytoplasm, while presenting more lamellopodia and filopodia. In contrast, M2-polarized macrophage have a rounded structure with actin located primarily around the nucleus^32^. These structural and morphological differences depend on rearrangement of the cytoskeleton, particularly actin filaments, which are phenotype and function specific^33^. Thus cytoskeletal actin arrangement enables both M1 and M2 functionalities^34^. As the cytoskeleton plays a critical role in numerous cellular processes, disruptions in its structure could lead to downstream effects on cell functionally, such as vesicle transport, phagocytosis, and migration^34–36^. Like many immune cell types in the body, exposure to microgravity promotes structural changes in macrophages. Interestingly, structural changes in macrophages are acute, occurring within seconds, and are transient. For example, within seconds of reaching microgravity in a suborbital rocket experiment (10^−4^ to 10^−5^ g), primary human macrophages undergo cytoskeletal changes with increases in both cell and nuclear volume and surface area^29^. These structural changes, which do not correlate to changes in lysosomes or actin matrix, are followed within minutes by adaptation back towards their pre-exposure state^29^. Following this type of short term suborbital flight and return to Earth’s normal 1-g, no detectable structural changes were observed^29^. This rapid (8–15 s) effect of microgravity on macrophage structure and morphology was also reported during parabolic flight by increased cell spreading^37^. When considering more long-term (72 h) effects on macrophage structure, the culture of U937 in simulated microgravity via a RWV bioreactor resulted in a decrease in actin protein expression accompanied by cytoskeletal disorganization and reduced cell proliferation^38^. Similar disorganization of cytoskeletal structural proteins, specifically, beta tubulin and vinculin, along with cell shape changes were also seen in the J-111 macrophage cell line under (RPM) simulated microgravity^39^ and true spaceflight^40^. Though they are used frequently to assess monocytic lineage cells^41^, it should be noted that the J-111 cell line is believed to be contaminated with HeLa cells^42^. For comparison, Tauber and colleagues demonstrated that following 11 days in space aboard the International Space Station, there was no significant change in the amount of f-actin and vimentin cytoskeleton in human primary macrophages, though they do report a reduction in the expression of cell adhesion molecule (ICAM-1) and no significant changes in CD18 expression^26^. As discussed later in the Adhesion and Migration section, CD18 is involved in cell-extracellular matrix binding and ICAM-1 (i.e., CD54) in cell–cell binding interactions. They also categorized immunostained f-actin and vimentin based on their micromorphological organization (i.e., strings, clusters, clouds) for comparison between 11-day and 30-day spaceflight samples and differentially oriented controls. While micromorphological changes in f-actin were less prevalent, there was a significant difference in vimentin micromorphology following 30-days in spaceflight in terms of the categorized cytoskeletal strings and clusters. However, the authors do note that these differences may result from the technical failure of their planned on-orbit fixation, as increased culture time and atmospheric reentry while unfixed are potential confounding variables.

In another spaceflight study of U937 cells, Paulsen et al. reported that the actin cytoskeleton was greatly disturbed and tubulin expression was disorganized^43^. They speculate that these results could indicate a dysfunction macrophage phenotype, one with reduced cytoskeletal-associated functionality, i.e., regarding migration and phagocytosis. Collectively, work has shown that in response to microgravity, macrophage structural changes appear to be cell type and duration dependent, with acute effects being observed followed by some degree of adaptation. As will be discussed later, there is a connection between cytoskeletal reorganization and direct modulation of gene expression^44–46^, therefore mechanical force transduction onto the nucleus, which occurs within seconds, could play a substantial role in the effects of microgravity seen on macrophages^47^. Indeed, structural changes often accompany functional changes, the ability of macrophages to adapt during microgravity is critical for human health during spaceflight.

### Adhesion and migration

Macrophage adhesion and migration are critical processes for maintaining physiological homeostasis. During macrophage recruitment to sites of local inflammation, macrophages transmigrate through the endothelial cell wall, basal membranes, and connective tissues. This transmigration is governed by proper cell–cell interactions between monocytes/macrophages and endothelial cells through integrins and selectins on the cell surface. The function of monocytes/macrophages to properly extravasate through the stepwise process of (1) rolling, (2) activation, (3) adhesion, and (4) transmigration is crucial to their success as early responders during the innate immune response^48^. Dysfunction of macrophage migratory processes is associated with increased susceptibility to infection^49,50^, therefore a great deal of work has been done to understand how microgravity exposure affects this process and can lead to negative effects on astronaut health. For example, CD18 is a necessary integrin for binding to the extracellular matrix and ICAM-1 is critical for monocyte/macrophage extravasation. Reported data surrounding the influence of microgravity on ICAM-1 and CD18 expression is varied. Previous work has demonstrated increased ICAM-1 expression in U937 macrophages in microgravity aboard both suborbital and parabolic flights, as well as in simulated microgravity in 2D clinostats^51^. Similarly, an increase was also seen under simulated microgravity in primary human M2-polarized macrophages in clinostat rotation. In contrast, however, murine BV-2 microglial cells, which are macrophages residing in the central nervous system, showed reduced ICAM-1 expression in both clinorotation and parabolic flight^51^. With data from real microgravity, Tauber et al. reported that primary human macrophages cultured aboard the International Space Station showed reduced expression of cell adhesion integrin ICAM-1, though CD18 did not significantly change between gravity test conditions^26^. Their immunofluorescence staining imaging and resulting image analysis data at 11-day and 30-day time points were compared to differentially oriented 1-g controls. Of note, changes in ICAM-1 expression under microgravity are reported to be rapid, reversible, and linked to cytoskeletal function^51–53^. In a more clinical evaluation, when assessing monocytes isolated directly from astronauts upon returning from spaceflight, expression levels of adhesion molecule CD62L, also known as L-selectin, was also found to be decreased^54^. CD62L is a necessary homing receptor for monocytes to circulate through peripheral tissues, playing a role in interactions with endothelial cells for the purpose of extravasation. A decrease in CD62L expression indicates a decrease in adhesive and tissue migration capabilities^54^. Interestingly, other researchers did not see a statistically significant difference in the expression of CD13, CD11, CD62L, or ICAM-1 in post-flight blood-derived monocytes^55^. CD13, also known as alanine aminopeptidase, is associated with homotypic cell adhesion in monocytes, though appears to potentially show activity in leukocyte migration as well^56^; CD11 is the alpha component of numerous integrins, including the previously mentioned CD18, that mediate monocyte/macrophage and other leukocyte adhesion^57^. Collectively, these studies show evidence that the expression of cell adhesion integrins is altered to varying degrees in both simulated and actual microgravity, and also varies with species and tissue-type. In addition to adhesion, migration as a parameter has also been shown to be markedly reduced in J-111 macrophage cells in spaceflight^40^. There appears to be a gravisensitive effect on the cell membrane affecting cell adhesion integrins as well as cell structural proteins, rather than a slower transcriptional or proteolytic mechanism^51^.

In addition to CD18 and ICAM-1, CD14 expression has also been investigated on macrophages in microgravity. A key aspect of macrophage immunological function is the recognition of foreign elements and their associated molecular domains, such as pathogen-associated molecular patterns (PAMPs). Pattern recognition receptors on the cell surface, such as CD14, bind these domains to trigger macrophage response. Tauber et al. reported that surface expression of CD14 was not significantly changed under microgravity compared to 1-g controls^26^. However, CD14 expression is dependent on the vector of gravity: samples under 1-g oriented “facing up” (apical surface up, basal surface down) had nearly 4X greater CD14 expression than those at 1-g “facing down” (basal surface up, apical surface down)^26^. The results from these studies provide insight into how the modulatory effect that microgravity has on adhesion and migration processes could be the cause of impaired immune function during spaceflight. Macrophage adhesion and mobility processes are critically important to maintaining physiological homeostasis, and how these processes are affected by spaceflight is important for astronaut health.

### Proliferation

The dynamics of macrophage proliferation is critically important in governing health and disease^58^. Tissue resident macrophages are thought to proliferate at low levels in steady-state conditions, but proliferation rates strongly increase after macrophage depletion or under inflammatory conditions or infection^59,60^. This normal process of varied proliferation is strongly linked to macrophage function in inflammation, therefore an understanding of how microgravity affects proliferation is important. The interest in the effect of spaceflight on macrophage proliferation dates back decades to work that showed increased proliferation of murine bone marrow-derived macrophages following 6–7 days of spaceflight^37^. More recently, Tauber et al. found that this observed proliferation was dependent on the vector of gravity, which macrophages “facing down” (basal surface up, apical surface down) undergoing higher levels of proliferation compared to samples “facing up” (apical surface up, basal surface down)^26^. They likened this apparent increase in macrophage proliferation to that seen under inflammatory conditions^58^. In contrast, time-dependent inhibition of U937 cell proliferation under RWV simulated microgravity has also been reported^38^. In relation to spaceflight, Hatton and colleagues reported a significant decrease in U937 cell proliferation in spaceflight compared to 1-g ground controls^61^. However, their in-flight 1-g centrifugation control also demonstrated significantly reduced proliferation compared to the ground control. As a possible mechanistic insight, it is proposed that microgravity exposure leads to downregulation of cdc25B, a phosphatase critical for G2/M phase transition in the cell cycle. It is believed that microgravity slows cell growth at the G2 phase in U937 cells, leading to decreased proliferation^38^. Interestingly, while proliferation was decreased, there was a lack of apoptosis of U937 cells under RWV simulated microgravity^62^. Given that microgravity induced upregulation of HSP70 (heat shock protein), a major stress protein that protects myeloid cells such as U937 from apoptosis, this is not entirely surprising^38^. Further support for enhanced cell viability under microgravity is seen through the mild transient expression of pro-apoptotic genes, which adapt back within minutes^47^. Wang and colleagues noted the induction of CCAAT-enhancer-binding protein (C/EBPβ) expression from primary mouse macrophages exposed to RWV simulated microgravity. This inflammation-regulated transcription factor modulates genes involved in monocyte/macrophage proliferation, as well as differentiation and immune response^63^. Collectively, this work shows that the proliferation of macrophages is affected by microgravity. Macrophage proliferation is a highly regulated process that is governed by in situ environmental conditions. Altered ability to regulate proliferation in response to inflammation or infection could result in an altered immune response and have health consequences in flight.

### Gene expression

Gene expression studies provide invaluable insight into molecular mechanisms by which cells and tissues are affected by microgravity. Genetic insight into macrophages increases knowledge of immune response and human health during spaceflight. Research on the genetic expression of macrophages in microgravity has yielded interesting findings. For example, transcriptomics analysis via microarray of U937 cells exposed to either parabolic flight (20 s of microgravity) or suborbital rocket (300 s of microgravity) revealed 1709 and 4667 differentially regulated transcripts, respectively^47^. The transcripts affected in parabolic flight included those associated with DNA replication and microtubule-based processes, while transcripts from suborbital flight included intracellular transport, mRNA processing, RNA and enzyme binding, post-translational regulation, cell cycle regulation, and cell division. The respective research group found that the trends in transcript expression following microgravity were reversed in hypergravity, suggesting that the cells underwent some degree of adaptation to the new gravitational conditions via rapid counter-regulation. They also noted quick adaptation of >98% of the transcripts to microgravity within the 300 s of suborbital rocket flight. Upon comparison of the affected transcripts between these different microgravity platforms, they identified 58 transcripts they speculate may be gravity-regulated^47^. Thiel and colleagues also assessed the transcriptional effects of ion channel inhibition in microgravity, as ion channels have been speculated to be involved in mechanical force transduction for cellular processes. However, they found that only 2.4% of microgravity-affected transcripts could be attributed to ion channel effects, potentially supporting their hypothesis that cytoskeletal force transduction may be a more significant effector on genetic regulation changes in microgravity. Shi et al. also evaluated the genetic expression of macrophages in microgravity via RNA-sequencing of murine bone marrow- derived macrophages. They reported a downregulation of genes associated with proliferation and differentiation under both rotary culture simulated microgravity as well as spaceflight. Compared to 1-g controls, rotary culture exhibited 1267 differentially expressed genes (DEGs), and spaceflight exhibited 2270 DEGs. Using gene ontology bioinformatics, the authors analyzed the DEGs for trends regarding specific biological processes, finding the noted downregulation in proliferation and differentiation pathways, as well as upregulation of apoptotic and RNA-splicing processes in spaceflight macrophages and upregulation in translation^28^. They also confirmed the downregulation of these markers of proliferation via RT-PCR. The myriad of effects seen in macrophage response to microgravity may result in part from altered signaling pathways, as Shi et al. also reported via gene expression analysis the downregulation of the RAS, ERK (also known as MAPK), and NFκB signaling pathways and upregulation of the p53 signaling pathway^28^. Of particular interest, they found that changes in the ERK and NFκB pathways could be rescued by respective agonizts. As these pathways represent many different biological processes within the cell, their altered regulation could create multifarious downstream effects. Another group assessed changes to NFκB signaling using a rat hindlimb unweighted model, which is another method presented for simulation of microgravity^64^. They noted significantly greater monocyte and macrophage adhesion to the aortic epithelium, though this was attributed to increased NFκB activation in the vascular epithelium rather than monocytes or macrophages^64^. Additionally, a different study reported no alterations in the ERK/MAPK pathway for monocytic U937 cells after 5 min of clinostat rotation and seemingly minimal effect on NFκB activation^65^. The variety of microgravity simulation or testing methods, cell types, assessment methodologies, and resulting data make it difficult to determine definitive signaling pathway reactions to microgravity. As a more focused example of genetic alterations from microgravity, other researchers showed that hypoxia-inducible factor 1 alpha (HIF-1α), which is speculated to play a role in monocyte/macrophage activation, is also differentially regulated in altered gravity, though HIF-1α-dependent gene expression adapted after 5 min of microgravity^30^. HIF is a supporter of the innate immune response and antibacterial action; macrophages experiencing hypoxia undergo a metabolic change to become more angiogenic, mitogenic, and pro-invasive^66^. Collectively, this points to the influence of microgravity on regulating HIF expression in macrophages and the broader impact on macrophage recruitment, activation, and response to inflammation.

### Cytokine production and immune response

Cytokine secretion has been shown to be affected by microgravity by numerous studies^27,37,38,54,67–70^. Several of the major cytokines of interest focused on in previous studies are IL-1, IL-6, IL-10, IL-12, and TNF-alpha. These cytokines are important mediators of the inflammatory response, and their decreased production has deleterious effects on the body’s ability to appropriately combat infection^71^. The normal function of these cytokines involves systemic inflammation, T- and B-lymphocyte activation and stimulation, as well as the induction of acute phase reaction proteins. IL-6 acts as both a pro-inflammatory and anti-inflammatory mediator as it can inhibit IL-1 and TNF-alpha, and stimulate IL-10. IL-1 and IL-12 are inflammatory cytokines which are involved in fever and epithelium adhesion factor expression, and T-cell differentiation, respectively. IL-10 is an anti-inflammatory agent that can reduce major histocompatibility complex class II (MHC-II) and co-stimulatory signal expression on macrophages, increased MHC-II expression being considered a hallmark of M1 polarization^72^. TNF-alpha is also related to systemic inflammation, stimulating fever, and increasing macrophage phagocytosis. Importantly, the effects on the production of these cytokines by macrophages experiencing microgravity are not always in agreement with the literature. Using IL-1 as an example, one study reported a decrease in IL-1^68^ after stimulation with radiolabeled phorbol ester while another reported near a complete lack of any IL-1 secretion by monocytes in a spaceflight study with stimulation of concanavalin A (ConA)^73^. Conversely, another study reported no difference in IL-1 production between monocytes in clinorotation and 1-g controls, with anti-CD3 stimulation^74^ while a study of U937 cells in RWV simulated microgravity by Maier et al. actually demonstrated increased IL-1 secretion with no stimulation^38^. This is consistent with a study by Chapes et al that reported increased IL-1 secretion, as well as TNF-alpha secretion, though in LPS stimulated bone marrow-derived macrophages in spaceflight versus 1-g LPS-stimulated controls^70^. The authors of another study demonstrated decreased TNF-alpha secretion and unchanged IL-1 secretion in LPS-stimulated RAW 264.7 cells in rotary culture. They also speculate that differences in their results compared to the previous work could be the result of different culture temperature conditions as they also used LPS stimulation^69^. Interestingly, a later study by Chapes et al. investigating peritoneal macrophages harvested from rats that underwent spaceflight found an increased secretion of TNF-alpha as well as IL-6^75^. Maier also reported an increase in TNF-alpha, IL-12, and IL-2, in addition to a large increase in IL-8 secretion compared to controls. A major aspect of the immune function of macrophages also comes from their expression of MHC class I and II. While many cells within the body basally express MHC-I, which can be upregulated in an inflammatory response, the role of macrophages as an antigen presenting cell for T-lymphocyte activation is affected through their expression of MHC-II. Paulsen et al. noted a statistically significant increase in MHC-I and decrease in MHC-II expression in U937 cells following 5 days in spaceflight. CD36, a scavenger receptor associated with phagocytosis, was also decreased under the influence of microgravity. The authors speculate that cytoskeletal disruption might contribute to the decrease in CD36 expression^43^.

As noted, differences in experimental design lead to differing results, as for macrophages in microgravity, IL-1 production has been shown to increase^38,54^, decrease^68^, or remain unchanged^27,69^. Similarly, IL-6 production has been reported to increase^27,63,75^ or decrease^37,54,67,69^ under the effects of microgravity; IL-10 increases^27^ or decreases^54^; IL-12 decreases^63,67,69^ or remains unchanged^27^. Likewise, TNF-alpha has been reported to increase^38,70,75^ or decrease^27,54,67,69^. Determining the causes of these differing results can prove difficult when not only cell sources and microgravity methods differ, but also macrophage stimulation. As seen above, numerous additives to media have been reported in the literature including macrophage phenotype stimulators such as LPS with or without IFN-γ^27,37,54,67,69,70^, ConA^73^, and IL-4^63^. As LPS, IFN-γ, and ConA are mitogens to drive an inflammatory cellular response (i.e., M1 macrophages) and IL-4 stimulates an M2 response, interstudy comparisons are further complicated. Attempting to standardize methods by focusing on commonly used cell types, preparation methods, and microgravity environment simulation/generation strategies will help to improve the consistency of results in the future.

### Reactive oxygen species

Regarding the immune response against viruses, bacteria, and fungi, macrophages produce reactive oxygen and nitrogen species (ROS, RNS, respectively) to degrade pathogen proteins. For example, M1 “pro-inflammatory” macrophages have upregulated expression of inducible nitrogen oxide synthetase (iNOS), a potent producer of reactive nitrogen species. The oxidative burst reaction resulting from the production of ROS is inhibited for macrophages while in microgravity^26,55,76,77^. Adrian et al. reported a reduction in ROS in microgravity that occurred rapidly and reversibly in the span of seconds using clinostat and parabolic flight models^76^. This significant reduction in ROS production by macrophages under clinostat simulated microgravity was independently corroborated by Brungs et al.^78^. Thiel et al. also reported immediate inhibition of ROS in a spaceflight study aboard the International Space Station followed by rapid adaptation (again, on the order of seconds) with extremely rare onboard real-time data to directly evince the gravisensitivity of oxidative burst. In the study, by Adrian et al., a delay in macrophage phagocytosis under clinorotation was also seen. Phagocytosis was significantly reduced after 30 min in clinorotation, but appeared to recover as no significant difference was seen compared to non-clinorotation controls at 60 and 180 min^76^. ROS are important for immunological defense and clearance of pathogens, as well as modulation of the immune response^79^ and differentiation^80^ of macrophages; this work demonstrates a reduction in ROS as a result of microgravity, reinforcing the notion that microgravity could increase susceptibility to disease through ROS reduction.

### Polarization

Given the influence macrophage phenotype can have on macrophage function, many microgravity studies have also considered the impact of microgravity on macrophage phenotype. Shi et al. found that microgravity inhibited the differentiation of macrophages from hematopoietic progenitor cells (in both rotary culture simulated microgravity and spaceflight) as well as their subsequent polarization into M1 and M2 phenotypes compared to those differentiated under normal gravity^28^. They also found that microgravity downregulated genes associated with macrophage polarization. This inhibition of macrophage polarization agrees with earlier work by Armstrong et al. in bone marrow-derived macrophages after spaceflight. They observed decreased expression of Galectin-3 (also known as MAC-2), a protein involved in macrophage activation in addition to cell adhesion and migration, as well as MHC-II, another marker of macrophage activation that is associated with macrophages’ role as antigen presenting cells. IL-6 secretion, associated with balancing M1 and M2 polarization, was also decreased in microgravity experienced macrophages which indicates an overall decrease in macrophage phenotype plasticity. Contrary to a decrease in macrophage polarization, Wang et al. reported upregulation of arginase 1, which is a consequence of increased C/EBPβ^63^. Arginase 1 is notable as a hallmark of alternatively activated macrophages, i.e., M2 macrophages, and is also important for cell proliferation as well as inhibiting the production of nitric oxide, a key signaling molecule of M1 macrophages^81^. As such, Wang et al.’s results could indicate the induction of an M2 phenotype resulting from microgravity. The work of Paulsen et al. contributes to the hypothesis of M2 polarization. They noted a distinct subtype of “activated and ameboid” BV-2 microglial cells with a simple rounded cell shape in their clinostat experiments^51^. Activated, ameboid microglia are considered to have undergone M2 polarization, and they function to block pro-inflammatory cytokines while promoting tissue repair and the release of neurotrophic factors, promoting healing in CNS tissue^82^. While M2 polarization is likely to occur as a result of microgravity, this work also demonstrates that macrophage polarization is likely to be downregulated or transient. Additional studies with genetic reference for M1 and M2 associated markers are needed to more adequately profile fluctuations in monocyte/macrophage expression as a result of microgravity.

## Conclusion

The literature on the effects of microgravity on monocyte/macrophages is diverse in its methodology. While rapidly occurring changes are seen in macrophages when exposed to microgravity, which often are observable within seconds of exposure^29,30,47,51,76–78^, the effects of microgravity are often reversible^51,76^ or quickly adapted to after the initial response^29,30,77^. Other work focused on the long-term effects of microgravity, using a time span of days to weeks^26,28,29,51,77,78^. The disparate use of numerous cell types and platforms when assessing microgravity can make direct comparisons difficult. However, the wide range of methods and cells tested can also be seen as an advantage when attempting to make more generalized statements regarding the effects microgravity has on macrophages. Seeing which measured variables demonstrate more robust and consistent responses across these different testing conditions and methods can help to highlight the most gravisensitive aspects of macrophage cellular biology. Several research groups have also focused on stepwise studies, utilizing several microgravity platforms to evaluate effects on macrophages, as well as shed some light on the possible validity of these simulated or alternative microgravity systems in replicating results from true spaceflight^39,40^. This stepwise approach is very helpful for validating observations and indicating where certain platforms may not be as reliable for approximating spaceflight. Continuation of this stepwise assessment utilizing multiple comparisons is necessary to move the field of microgravity biology forward. However, the variety of cell types used across experiments, which range from primary human monocytes, to primary murine macrophages, to histiocytic lymphoma cells, to tissue-specific polarized macrophages, presents another pressing issue in comparing results across studies. Indeed, authors have indicated that differing cell types even within their own studies contribute to contrasting results^51^. If comparability of research results to in vivo human is desired, the use of primary human cell sources is optimal^26^. In order to attain comparable results, multiple microgravity modalities, including true spaceflight as a golden standard comparison if possible, should be considered. Careful consideration of cell source given the intended research focus should also be conducted to ensure research relevancy and adequate comparisons.

## Data Availability

All data in this paper can be found in previous publications cited within the text. No new datasets were generated or presented in this manuscript.
